# Limb length discrepancy and gait parameters of amateur football players in Lagos State, Nigeria

**DOI:** 10.17159/2078-516X/2020/v32i1a8029

**Published:** 2020-01-01

**Authors:** A K Akodu, O A Akindele

**Affiliations:** Department of Physiotherapy, College of Medicine, University of Lagos, Nigeria

**Keywords:** athletes, real leg length, step length, spatial distance

## Abstract

**Background:**

Football is a widely played sport globally. Limb length discrepancies have been found to be common among football players and these may lead to abnormal gait.

**Objectives:**

The purpose of this study was to determine the relationship between limb length discrepancy (LLD) and gait parameters of amateur football players in Lagos State, Nigeria.

**Methods:**

Eighty-nine amateur football players participated in the cross-sectional study. They were recruited from different stadia in Lagos State. Limb length and gait parameters were measured using tape and footprint measurements.

**Results:**

The prevalence of real limb length discrepancy among the participants was 75% (n = 67). The results of this study showed that the right leg was the shorter leg in 60% (n = 53) of the participants. There was no significant correlation between gait parameters and limb length measurement.

**Conclusion:**

Although limb length discrepancy is common among amateur football players in Lagos State, the relationship between limb length and gait parameters is weak.

Playing football rigorously while growing up may lead to the appearance and development of a real limb length discrepancy (LLD). ^[1]^More precisely, the observations designate that growth is slower in the lower limb used to kick the ball and may lead to LLD. The weight of the ball and an intensive careful play regimen may be aggravating factors.^[1]^ Abnormalities in gait pattern can be a consequence of limb length discrepancy of two cm or more, resulting in an increase in muscle activity, heart rate and oxygen consumption.^[2]^Disparity in limb length is usually connected to the development of gait abnormalities which can result in degenerative arthritis of the lower extremity and lumbar spine.^[3]^

A gait cycle can be defined as a time interval between the heel strike of an extremity and when the same heel contacts the ground again.^[4]^During locomotion, the gait cycle of a healthy individual is high but the gait of an unhealthy individual displays deviation from its normal cycle.^[5]^ The more time a person spends training, the more probable their gait will be less periodic and less harmonic.^[6]^ This can be explained by the fact that training beyond a critical level could lead to musculoskeletal reconstruction that has not been recognised by the locomotor centres in the neural network, resulting in a gait that is less well-controlled.^[6]^

Amateur football is a compound sport and its performance is based on physical, physiological and psychological aspects. Due to the paucity of data on limb length discrepancy and gait parameters of amateur football players in the environment, this study was conducted to determine the correlation between the aforementioned variables in amateur football players in Lagos State, Nigeria.

## Methods

This cross-sectional survey was carried out between May and October 2019. The study involved 89 amateur football players recruited from six football teams in different stadia in Lagos State and used a non-probability sampling technique (Purposive Sampling technique).This sampling technique also used in the study of Sajjan,^[7]^ was adopted because of the small sample that exists among the registered amateur football clubs involved in this study. Only registered amateur football players were included in the study. The football players who had stopped playing football but who participated in other sports and had a previous or current sport injury were excluded.

Before conducting this research, written permission was obtained from the Health Research and Ethics committee of the College of Medicine at the University of Lagos (approval number: CMUL/HREC/06/19/551). The reason for the study was clearly explained to the participants. They were also assured of the privacy of their responses. Each participant gave written informed consent before the start of the study.

### Assessment of limb length discrepancy

The tape measurement method (TMM) was used for measuring limb length discrepancy. This was undertaken using two examiners and had an Intraclass Correlation Coefficient (ICC) of 0.922 and 0.990 with an excellent intra-rater agreement of 0.990 and 0.985 respectively. ^[8]^

The pelvis was aligned against the back of the plinth, and the distance between the medial malleoli of each tibia was measured.^[1]^ Using a tape measure, the researcher assessed the distance between the anterior superior iliac spine to the medial malleolus (real length) of the leg and from the umbilicus to the lateral malleolus (apparent length) of the leg. A comparison of both sides was done.^[9]^ A limb length discrepancy (LLD) of 1.1 cm and above was classified as abnormal.

### Assessment of gait parameters

Gait parameters were measured by covering a 10 m walkway with white cardboard. Participants placed both feet in a tray of talcum powder. They were then instructed to walk normally along the pathway, starting with the right foot. The researcher advised the participants to look ahead and continue the same walking pace until the end of the cardboard ^[10]^.

An assessment of distance parameters (step and stride length) was done with a metal ruler and recorded in centimetres. The step length was assessed as a line at right angles to the heel’s reference points and its intersection with the ipsilateral line of progression. Stride length was assessed as the spatial distance between the heel’s reference points of one foot on the ipsilateral side. The base of the gait was measured as the spatial distance between the heel’s reference points and the opposite ipsilateral line of progression measured in centimetres ^[1]^. The gait was assessed by only one examiner. However, other examiners found the technique to be reliable, as evidenced by Pearson’s r correlation coefficients ranging from 0.92 to a perfect correlation of 1.00 ^[11]^.

### Statistical analysis

The data were analysed using IBM SPSS (Statistics for Windows, Version 25.0. Armonk, NY: IBM Corp) and summarised using the descriptive statistics of mean, standard deviation, frequency and percentage. Pearson’s correlation for parametric variables was used to determine correlation between LLD and gait parameters. The authors accepted an alpha level of 0.05.

## Results

The participants were between the ages of 14 and 31 years, with a mean age of 22 ± 3 years. The mean body mass index (BMI) of the participants was 21.5 ± 1.5 kg/m^2^ (Table1).

The mean values of the right real limb length and left apparent limb length were 96.5 ± 4.0 cm, and 102.3 ± 8.4cm respectively (Table 2). Twenty-five percent of the participants (n = 22) had normal limb length. In 41% of the participants (n =36) the right leg was longer than the left leg (Table 2).

The mean of the step length, stride length and the base of support of the participants was 36.3 ± 10.9 cm, 84.4 ± 18.8 cm and 15.6 ± 6.6 cm respectively (Table 2).

The correlation between limb length measurement and the physical properties of the participants are shown in Table 3. There was a significant but weak positive correlation (r =0.23, p=0.03) between age and right real limb length. All limb length measurements had a significant positive correlation with height. There was a significant moderate positive correlation between height and right real limb length(r = 0.41, p=0.001) and height and apparent limb length (r = 0.51, p=0.001) (Table 3). There was a significant moderate positive correlation between weight and real limb length measurement (right) (r = 0.41, p=0.001).

There were no significant correlations of any of the limb length measurements and gait parameters (Table 4).

## Discussion

This study was undertaken because the literature on the leg length discrepancy and gait in amateur football players is sparse. The authors have shown that 75% of the participants had limb length discrepancies, with 59% having a shorter right leg. The shorter limb is often the kicking leg ^[1]^.

The walking pattern of athletes was no different to that of non-athletes, as seen in subjects used in the study by Leroy et al.^[12]^. However, there may be differences depending on the sport played. For example, the step length has been found to vary among football players, and playing football indicates differences in the locomotion pattern between the right and left lower limbs.^[12]^This study has confirmed this since there was no relationship between the limb length and gait parameters.

A previous study showed that playing football intensively before or during the period of growth may encourage the appearance of a real limb length discrepancy. ^[1]^ More precisely, growth is slowed in the lower limb used to kick the football which may lead to a leg length discrepancy of up to 17 mm.^[1]^Among players who began their football career before the age of 13 years, 96% had the kicking leg as the shorter limb. This pattern was observed in only 53% of the players who started playing football later. ^[1]^

## Conclusion

Based on the results of this study, it can be concluded that there was high prevalence of the real limb length discrepancy among the participants. It is recommended that amateur football players with limb length discrepancy should be encouraged to train specifically and use proper footwear to prevent poor gait parameters.

## Figures and Tables

**Table 1:** Physical characteristics of the participants (n=89)

Characteristic	Frequency (n)	Percentage (%)
**Age groups (years)**		
≤20	24	27
21–25	58	65
26–30	5	6
31–35	2	2
Mean	22 ± 3	

**Height (m)**		
1.40–1.50	1	1
1.51–1.60	2	2
1.61–1.70	20	23
1.71–1.80	66	74
Mean	1.74 ± 0.06	

**Weight (kg)**		
51–55	3	3
56–60	10	11
61–65	30	34
66–70	37	42
71–75	9	10
Mean	65.2 ± 1.5	

**Body mass index (kg/m** **^2^** **)**		
Under 18.50	1	1
18.50–24.90	86	97
25.00 – 29.90	2	2
Mean	21.5 ± 1.5	

Data are expressed as a frequency of the total participants per characteristic. Where applicable data are expressed as mean ± SD. Body Mass Index (BMI) range; underweight (< 18.5 kg/m^2^), normal weight (18.5 to 24.9 kg/m^2^), overweight (25.0 to 29.9 kg/m^2^).

**Table 2:** Gait parameters and limb length measurement of the participants (n= 89)

Variables	Mean ± SD	Frequency (n)	Percentage (%)
**Gait parameters**			
Step length (cm)	36.3 ± 10.9		
Stride length (cm)	84.4 ± 18.8		
Base of support (cm)	15.6 ± 6.6		

**Limb length measurements**			
Real limb length right (cm)	96.5 ± 4.0		
Real limb length left (cm)	97.5 ± 6.6		
Normal real limb length difference		22	25
Abnormal real limb length difference		67	75

Apparent limb length right (cm)	100.9 ± 4.3		
Apparent limb length left (cm)	102.3 ± 8.4		
Normal apparent limb length difference		15	17
Abnormal apparent limb length difference		74	83

**Longer limb**			
Right leg		36	41
Left leg		53	59

Data are expressed as a frequency of the total participants per variable group or expressed as mean ± SD. An apparent or real limb length difference of < 1.1 cm was classified as normal; a difference ≥ 1.1 cm was classified as abnormal.

**Table 3:** Correlation between limb length measurement and anthropometric variables of the participants (n=89)

	Real length right	Real length left	Apparent length right	Apparent length left
**Age (years)**				
r	0.23	−0.06	0.08	−0.07
95% CI	0.02 to 0.42	−0.15 to 0.26	−0.13 to 0.28	−1.14 to 0.27
p value	0.03*	0.58	0.48	0.52

**Height (m)**				
r	0.41	0.22	0.51	0.23
95% CI	0.22 to 0.57	0.01 to 0.41	0.34 to 0.65	0.02 to 0.42
p value	0.001*	0.04*	0.001*	0.03*

**Weight (kg)**				
r	0.41	0.26	0.37	0.17
95% CI	0.22 to 0.57	0.05 to 0.44	0.18 to 0.54	−0.04 to 0.37
p value	0.001*	0.01*	0.001*	0.12

**BMI (kg/m** **^2^** **)**				
r	0.01	0.05	−0.14	−0.06
95% CI	−0.20 to 0.22	−0.16 to 0.26	−0.07 to 0.34	−0.15 to 0.26
p value	0.91	0.63	0.18	0.56

* Significant at p<0.05.

r, Pearson correlation coefficient; CI, confidence interval; BMI, body mass index

**Table 4:** Correlation between limb length measurement and gait parameters of the participants (n=89)

	Real length right	Real length left	Apparent length right	Apparent length left
**Step length (cm)**				
r	−0.09	−0.14	0.06	−0.07
95% CI	−0.29 to 0.12	−0.34 to 0.07	−0.15 to 0.26	−0.27 to 0.14
p value	0.38	0.20	0.60	0.51

**Stride length (cm)**				
r	0.05	−0.08	0.02	0.04
95% CI	−0.16 to 0.26	−0.28 to 0.13	−0.19 to 0.23	−0.17 to 0.25
p value	0.65	0.49	0.85	0.73

**Base of support (cm)**				
r	−0.11	−0.15	−0.07	−0.03
95% CI	−0.31 to 0.10	−0.35 to 0.06	−0.27 to 0.14	−0.24 to 0.18
p value	0.32	0.15	0.54	0.78

r, Pearson correlation coefficient; CI, confidence interval
